# Plantar Stimulations during 3-Day Hindlimb Unloading Prevent Loss of Neural Progenitors and Maintain ERK1/2 Activity in the Rat Hippocampus

**DOI:** 10.3390/life11050449

**Published:** 2021-05-17

**Authors:** Anna S. Berezovskaya, Sergey A. Tyganov, Svetlana D. Nikolaeva, Alexandra A. Naumova, Boris S. Shenkman, Margarita V. Glazova

**Affiliations:** 1 Sechenov Institute of Evolutionary Physiology and Biochemistry Russian Academy of Sciences, 194223 St. Petersburg, Russia; berezovskaia.annaS@gmail.com (A.S.B.); sveta.nikolaeva@gmail.com (S.D.N.); anaumova07@gmail.com (A.A.N.); 2 Institute of Biomedical Problems Russian Academy of Sciences, 123007 Moscow, Russia; sentackle@yandex.ru (S.A.T.); bshenkman@mail.ru (B.S.S.)

**Keywords:** simulated microgravity, hippocampus, neurogenesis, doublecortin, Ki67, ERK1/2, NR2B

## Abstract

Adult neurogenesis is a flexible process that depends on the environment and correlates with cognitive functions. Cognitive functions are impaired by various factors including space flight conditions and reduced physical activity. Physically active life significantly improves both cognition and the hippocampal neurogenesis. Here, we analyzed how 3-day simulated microgravity caused by hindlimb unloading (HU) or dynamic foot stimulation (DFS) during HU can affect the hippocampal neurogenesis. Adult Wistar rats were recruited in the experiments. The results demonstrated a decrease in the number of doublecortine (DCX) positive neural progenitors, but proliferation in the subgranular zone of the dentate gyrus was not changed after 3-day HU. Analysis of the effects of DFS showed restoration of neural progenitor population in the subgranular zone of the dentate gyrus. Additionally, we analyzed activity of the cRaf/ERK1/2 pathway, which is one of the major players in the regulation of neuronal differentiation. The results demonstrated inhibition of cRaf/ERK1/2 signaling in the hippocampus of HU rats. In DFS rats, no changes in the activity of cRaf/ERK1/2 were observed. Thus, we demonstrated that the process of neurogenesis fading during HU begins with inhibition of the formation of immature neurons and associated ERK1/2 signaling activity, while DFS prevents the development of mentioned alterations.

## 1. Introduction

Adult neurogenesis was firstly discovered by Joseph Altman and Gopal D. Das in 1965 [1]. There are two main neurogenic niches in the adult mammalian brain—the subgranular zone of the dentate gyrus and the subventricular zone. Adult hippocampal neurogenesis is a flexible process that depends on many different factors [2]. A physically active life and an enriched environment stimulate neurogenesis and correlate with improved cognition [2]. Conversely, indolence or immobility caused by traumas or diseases can negatively affect both neurogenesis and cognitive functions [3,4]. It was reported that long-term space flight not only affects musculoskeletal, cardiovascular, and other peripheral systems, but also leads to morphological and functional changes in the central nervous system (CNS) both in humans and animals [5,6,7]. There are no data about the functional state of adult neurogenesis during or after space flight, but a few papers demonstrated negative effects of long-term hindlimb unloading (HU) on the hippocampal neurogenesis of rodents [8,9]. HU is a widely used model of simulated microgravity that reproduces such changes as reduced motor activity, atrophy of hindlimb muscles, and cephalic fluid shift [10,11]. To prevent muscle atrophy, dynamic foot stimulation (DFS) of the plantar surface was invented firstly for astronauts [12] and then for rodents [13]. In our previous study, we demonstrated that attenuation in the activity of glutamatergic system of the hippocampus in rats after 3-day HU was compensated by DFS [14]. Many endogenous factors affect adult hippocampal neurogenesis, including glutamate, which participates in the regulation of neuronal differentiation [15,16]. It is known that physical exercises stimulate expression and release of neurotrophins, such as brain-derived neurotrophic factor (BDNF), from the muscles into the blood circulation, and thus stimulate hippocampal neurogenesis [17,18]. On the other hand, BDNF also stimulates glutamate secretion from the neurons of the hippocampus [19,20] and upregulates expression of vesicular glutamate transporters 1 and 2 (VGLUT1/2) in the hippocampal neurons [21]. Expression of VGLUTs directly reflects glutamatergic neurotransmission [22] and we supposed that a significant decrease in the expression of VGLUT1/2 in the hippocampus after 3-day HU [14] may result from muscle disuse. Based on published and our data, we hypothesized that attenuation of glutamatergic system in the hippocampus and decreased motor activity can affect the hippocampal neurogenesis during the first days of HU. Here, we analyzed the hippocampus of 3-day HU rats and rats with DFS applied during HU to verify if neurogenesis can be affected by short-term HU and/or DFS. We have shown beneficial effects of DFS applied during the first days of muscle disuse, indicating the importance of starting physical exercise as soon as possible to prevent the development of brain disturbances in astronauts and bedridden patients.

## 2. Materials and Methods

*Animals*. Adult male Wistar rats (130–160 g; IBCh RAS, Pushchino, Moscow, Russia) were recruited in the experiments. The rats were housed in individual cages at a 12 h/12 h light–dark cycle with free access to water and food. All procedures were conducted in accordance with EC Directive 86/609/EEC for animal experiments and approved by the Biomedical Ethics Committee of the Institute of Biomedical Problems.

*Experimental design*. The experimental procedures have been previously published and, here, we continue the analysis of 3 day HU rats and rats with DFS applied during HU [14]. To simulate microgravity, we used the hindlimb unloading (HU) model according to Morey-Holton [10]. To stimulate cutaneous mechanosensory receptors as occurs with natural locomotion, during HU, the dynamic foot stimulation (DFS) of the plantar surface was applied according Kyparos et al. [13]. HU procedure: an elastic bandage was wound around the tail and attached with a swivel to a metal rod at the top of the cage. The suspension angle was approximately 30°, which allowed the rats to move around the cage only using their forelimbs. DFS procedure during 3-days of HU: custom-built boots with movable plastic plates contacting with the sole of the foot were attached to both hindlimbs. Pressure at 104 mmHg was applied cyclically by the air bladder with a 1 s inflation/1 s deflation for a total of 20 min followed by a 10 min rest period during 4 h every day from 10:00 to 14:00. In the experiments, there were three groups: C group (*n* = 12)—vivarium control; HU group (*n* = 12)—3-day hindlimb unloading; and DFS group (*n* = 12)—4 h dynamic foot stimulation of the plantar surface each day during HU. The rats were anaesthetized with an intraperitoneal injection of tribromoethanol (240 mg/kg; # T48402, Sigma-Aldrich, St. Louis, MO, USA) and perfused with 4% paraformaldehyde for immunohistochemical study (*n* = 4 for each group), or decapitated and ventral hippocampi were dissected for left and right parts and homogenized for Western blot assay (*n* = 8 for each group, left parts) or collected in TRI Reagent (#T9424, Sigma-Aldrich, St. Louis, MO, USA) for RNA isolation (*n* = 4 for each group, right parts).

*Immunofluorescence*. The cut sections (10 μm) containing the hippocampus were incubated overnight at room temperature with primary antibodies: anti-Ki67 (1:200; #AB9260, Millipore, Burlington, MA, USA) and anti-DCX (1:300; #4604, Cell Signaling Technology, Danvers, MA, USA). Then, the sections were washed in PBS and incubated with anti-rabbit Alexa488 (IF; 1:1000; #A11008, ThermoFisher, Waltham, MA, USA). Cell nuclei were stained by DAPI (1:2000, #28718-90-3, Sigma-Aldrich, St. Louis, MO, USA). Analysis was performed using the Leica AF7000 fluorescent microscope (Leica Microsystems GmbH, Wetzlar, Germany).

*Western blot*. The hippocampi were dissected and homogenized in lysis buffer (20 mM Tris, pH 7.5; 1 mM EDTA; 1 mM EGTA; 150 mM NaCl; 1% Triton X-100) with protease inhibitors (#4693116001, Sigma-Aldrich, St. Louis, MO, USA) and phosphatase inhibitor cocktail (#4906837001, Sigma-Aldrich, St. Louis, MO, USA). The protein concentrations were determined by Bio-Rad protein assay (bovine serum albumin standard, #5000002, Bio-Rad Laboratories Inc., Hercules, CA, USA). Equal amounts of protein (15 µg per line) in sample buffer (#1610738, Bio-Rad Laboratories Inc., Hercules, CA, USA) were denatured at 95 °C for 5 min and separated on 10% or 12% acrylamide gels using Mini-PROTEAN tetra Handcast System (Bio-Rad Laboratories Inc., Hercules, CA, USA). The proteins from the gel were transferred to a nitrocellulose membrane (#sc-3718, Santa Cruz Biotechnology, Dallas, TX, USA) blocked in 3% non-fat milk in Tris buffer and incubated overnight with primary antibodies against the following: cRaf (1:1000; #9422, Cell Signaling Technology, Danvers, MA, USA); p-cRaf (1:1000; #9421, Cell Signaling); ERK1/2 (1:1000; #9102, Cell Signaling); pERK1/2 (1:1000; #4376, Cell Signaling); NR2B (1:1000; #ab65875, Abcam, Cambridge, UK); and GAPDH (1:2000; # ab8245, Abcam, Cambridge, UK). Then, the membranes were incubated with secondary antibodies, anti-rabbit (1:40,000; #A0545, Sigma-Aldrich) or anti-mouse (1:40,000; #A9044, Sigma-Aldrich), followed by chemiluminescent detection by SuperSignal@West Dura Extended Duration Substrate (#34075, ThermoFisher Scientific, Waltham, MA, USA). The signals were captured by ChemiDoc MP Imaging System (#12003154, Bio-Rad) and analyzed using ImageJ software.

*Quantitative Real-Time PCR*. Total RNA was isolated from the hippocampi (*n* = 4 for each groups), using TRI Reagent (#T9424, Sigma-Aldrich, St. Louis, MO, USA), and first-strand cDNA synthesis was performed with Oligo (dT) primers and RevertAid First Strand cDNA Synthesis Kit (#K1622, ThermoFisher Scientific) and 0.5 μg total RNA. qPCR was performed on Applied Biosystems 7500 real-time PCR system (ThermoFisher Scientific) using a qPCRmix-HS SYBR (#PK156, Eurogen, Russia). Primers: *Sox2* (NM_001109181.1) forward—5′- AGG AGC AGC TGG GCT ACC -3′, *Sox2* reverse—5′- CTG CGA GTA GGA CAT GCT GTA -3′; *cyclin D1* (NM_171992.4) forward—5′- AAG GAG ACC ATT CCC CTG AC -3′, *cyclin D1* reverse—5′- TCT GGC ATT TTG GAG AGG AAG -3′; *GAPDH* (NM_017008.4) forward—5′-TCC CTC AAG ATT GTC AGC AA-3′, *GAPDH* reverse—5′-AGA TCC ACA ACG GAT ACA TT-3′. Relative fold expression of genes was calculated in Microsoft Excel by the 2-ΔΔCt method.

*Evaluation of sections and statistical analysis*. Four rats per group were taken for immunofluorescence assay and eight rats per group were taken for Western blot assay. The sections of control and experimental groups were processed for immunostaining, and Ki67 and DCX positive cells were counted in the hippocampus. Five sections of the hippocampus were analyzed for each animal per group and the data for every rat on graphs are presented as median per slice. Statistical analysis was done with a nonparametric Kruskal–Wallis test followed by Dunn’s post-hoc test using GraphPad Prism 8.2.1. (GraphPad Software, San Diego, CA, USA). Values were considered statistically significant for *p* < 0.05. In all graphs, values are expressed as median with interquartile range for both cell counting on immunofluorescent images and Western blot assay.

## 3. Results

### 3.1. DFS during 3-Day Hindlimb Unloading Prevents Loss of DCX Positive Neural Progenitors

**Figure 1:**
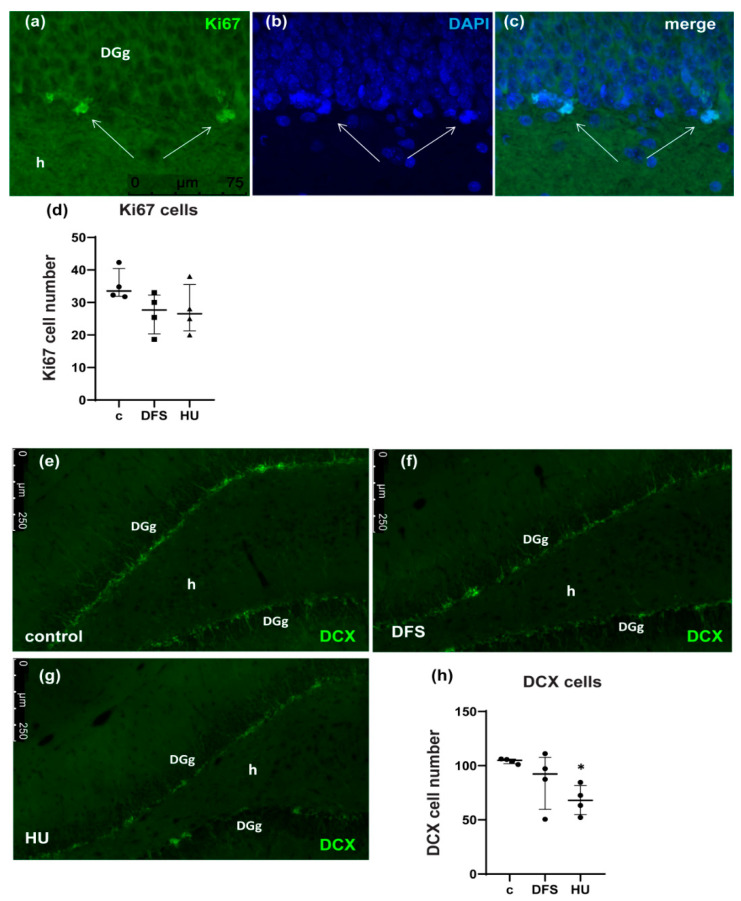
DFS during 3-day hindlimb unloading prevents loss of DCX positive neural progenitors. (**a**–**c**) Ki67 immunostaining of the dentate gyrus of control rats. Immunofluorescente images of the dentate gyrus of control, HU, and DFS rats are presented in the Supplementary Material (Figure S1). DAPI – 4’, 6-Diamidino-2-Phenylindole; DGg—the granular layer of the dentate gyrus; h—hilus. (**d**) Number of Ki67 positive cells in the subgranular zone of the dentate gyrus. Five sections of the hippocampus from each rats were analyzed (*n* = 4 rats per group). Data are shown as median with interquartile range. (**e**–**g**) DCX immunostaining of the dentate gyrus of control rats (control) (**e**), hindlimb unloaded rats with dynamic foot stimulation (DFS) (**f**), and hindlimb unloaded rats (HU) (**g**). DGg—granular layer of the dentate gyrus; h—hilus. (**h**) Number of Ki67 positive cells in the subgranular zone of the dentate gyrus. Five sections of the hippocampus from each rats were analyzed (*n* = 4 rats per group). Data are shown as median with interquartile range. * *p* < 0.05 vs. control. c—control, DFS—the rats with dynamic foot stimulation during 3-day hindlimb unloading; HU—3-day hindlimb unloading.

Firstly, we analyzed proliferation in the subgranular zone of the dentate gyrus. As a marker for proliferated cells, we used Ki67 and counted Ki67 positive cells on five slices of the hippocampus of each rat in all groups. The obtained results did not reveal any changes in the number of Ki67 positive cells between all groups (Figure 1a–d; Figure S1). Expression of Sox2 mRNA (SRY (sex determining region Y)-box 2), which controls proper self-renewal of neural stem cells [23], in all groups was the same (Figure S2a); expression of Cyclin D1 mRNA, which regulates cell cycle progression [24], was not changed as well (Figure S2b). However, analysis of doublecortin (DCX) expression (Figure 1e–g), which is a marker of immature migrating neurons [25], demonstrated a significant decrease in the number of DCX positive cells after 3-day HU, but there was no difference between control and DFS groups (Figure 1h; c vs. HU *p* = 0.0372; c vs. DFS *p* = 0.4786; HU vs. DFS *p* = 0.7179).

### 3.2. Dynamic Foot Stimulations Rescue Activity of CRAF/ERK1/2

**Figure 2:**
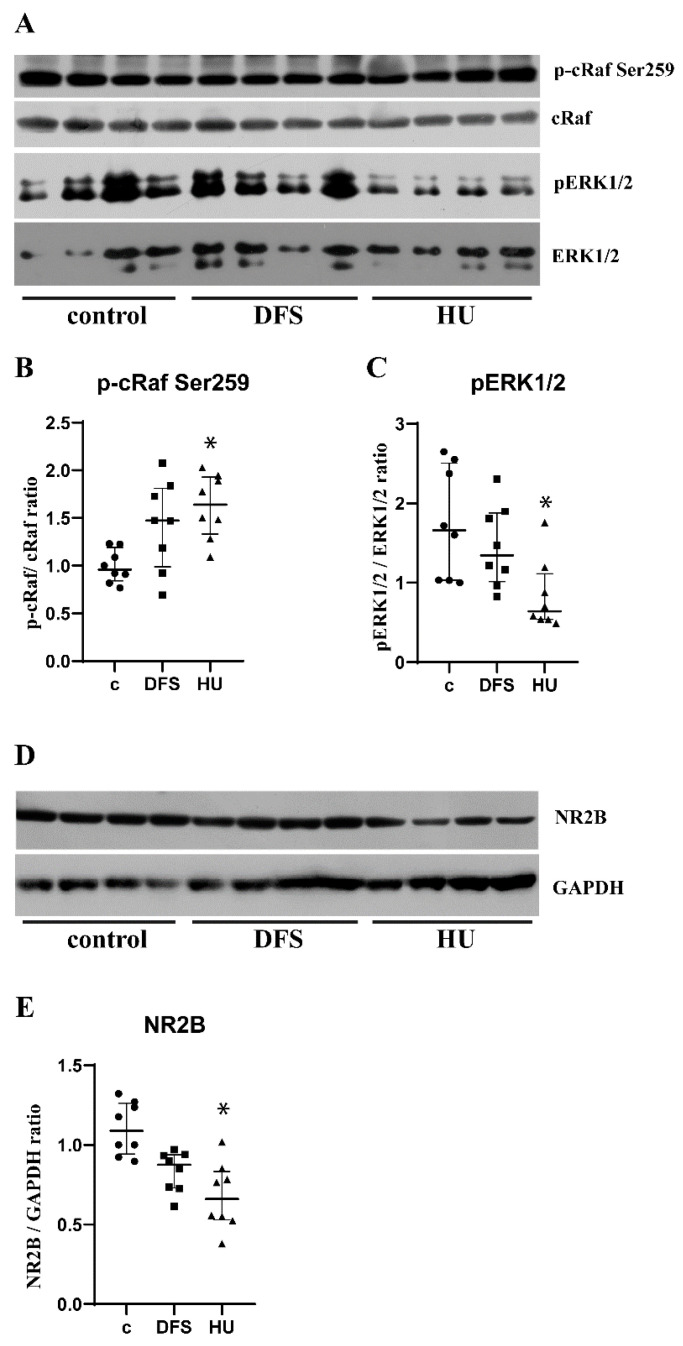
Dynamic foot stimulations rescue activity of cRaf/ERK1/2. (**A**) Representative images of Western blots of p-cRaf, cRaf, pERK1/2, and ERK1/2. (**B**) Analysis of cRaf phosphorylation at Ser259 by calculation of the ratio of p-cRaf to total cRaf. (**C**) Analysis of ERK1/2 phosphorylation at Thr202/Tyr204 by calculation of the ratio of pERK1/2 to total ERK1/2. (**D**) Representative images of Western blots of NR2B and GAPDH. (**E**) Analysis of NR2B expression by calculation of the ratio of NR2B to GAPDH; c—control, DFS—the rats with dynamic foot stimulation during 3-day hindlimb unloading; HU—3-day hindlimb unloading. *n* = 8 rats per group. Data are shown as median with interquartile range. * *p* < 0.05 vs. control. (Original Western Blots figure see Supplementary Materials).

One of the major players in the neuronal differentiation is ERK1/2 signaling [26]. Therefore we analyzed phosphorylation of cRaf at inhibitory site Ser259, and phosphorylation of downstream kinase ERK1/2 [27]. Our data demonstrated significantly increased phosphorylation of cRaf at Ser259 in the hippocampus of HU rats (Figure 2A,B; c vs. HU *p* = 0.0056; c vs. DFS *p* = 0.143; HU vs. DFS *p* = 0.773) accompanied by decreased phosphorylation of ERK1/2, while DFS successfully prevented ERK1/2 signaling deactivation (Figure 2A,C; c. vs HU *p* = 0.0362; c. vs. DFS *p* > 0.999; HU vs. DFS *p* = 0.137).

Activity of ERK1/2 is regulated by NR2B subunit of the NMDA receptors [28]. We subsequently analyzed expression of NR2B and revealed a decrease of NR2B expression in the hippocampus of HU rats, while in DFS group, the expression of NR2B remained at the control level (Figure 2D,E; c vs. HU *p* = 0.0021; c vs. DFS *p* > 0.0777; HU vs. DFS *p* = 0.7297).

## 4. Discussion

The adult hippocampal neurogenesis is a complex process, which starts with proliferation and is followed by several differentiation stages if the early postmitotic progenitors survive [29]. Previously, a few published data demonstrated that long-term HU leads to inhibition of the hippocampal neurogenesis [8,9]. However, there are no data showing what is impaired primarily during HU: proliferation or early postmitotic differentiation or both? Our data revealed a decrease in DCX positive cell number in the dentate gyrus without any alteration in the number of Ki67 positive proliferating cells after 3-day HU. These facts point to early postmitotic progenitors as a critical chain link in the control of neurogenesis.

Different intracellular signaling molecules tightly participate in the regulation of neuronal proliferation and differentiation such as PKA, Akt, and cAMP response element-binding protein (CREB). Previously, we demonstrated that 3-day HU did not affect the activity of PKA, but did activate Akt/GSK3b/CREB dependent cell survival. Additionally, activation of CREB mainly reflected the activation of the granular cells of the dentate gyrus and the pyramidal cell of the CA3 and CA1, but not the cells of the subgranular zone [14]. Further, one of the factors that regulate neuronal differentiation is ERK1/2 kinase [26]. Moreover, Lee with co-authors demonstrated the activation of ERK1/2 in DCX positive neural progenitors in the subgranular zone of the dentate gyrus that promotes its survival and differentiation [30]. In our experiments, we observed that activity of ERK1/2 signaling was significantly decreased in line with a decrease in DCX expression. Thus, we demonstrated that the process of neurogenesis fading during short-term HU begins with a decrease in neuronal differentiation and the associated ERK1/2 signaling activity.

In addition to ERK1/2, there are other participants involved in the regulation of adult hippocampal neurogenesis, of which glutamate regulates migration of neural progenitors and neuronal differentiation [15,16]. Moreover, published data showed activation of ERK1/2 signaling by glutamate in an NR2B dependent manner [28]. Previously, we have demonstrated that depression of the glutamatergic system in the hippocampus of 3-day HU rats [14] can also affect the activity of ERK1/2 and, further, the maturation of neural progenitors. Additionally, here, our data revealed a decrease in NR2B expression in the hippocampus of HU rats.

On the other hand, physical activity induces and maintains neurogenesis in adults via upregulation of neurotrophins, mainly brain derived neurotrophic factor (BDNF) and its receptors TrkB in the brain [17]. In turn, ERK1/2 is one of the main intracellular signaling that mediate the effects of the BDNF/TrkB pathway [31]. Here, we used DFS to stimulate the sensory receptors in the soles of the rat’s feet, which mimics natural locomotion, prevents muscle atrophy, and restores protein synthesis during the first days of HU [13,32]. Indeed, our data demonstrated that DFS applied during short-term HU successfully prevented loss of DCX positive progenitors and normalized expression of NR2B receptors and activity of ERK1/2. Summarizing the obtained results and our previously published data [14], we conclude that a decrease in glutamate signaling during shot-term HU leads to inhibition of ERK1/2 and decreased neurogenesis, while DFS eliminates the development of mentioned alterations. Additionally, we suppose that DFS may restore activity of ERK, probably, by stimulation of the expression of neurotrophins, but these mechanisms remain to be elucidated further.

## Data Availability

Not applicable.

## Supplementary Materials

The following are available online at https://www.mdpi.com/article/10.3390/life11050449/s1, Figure S1: Representative pictures of Ki67 immunostaining (green) in the subgranular zone of the dentate gyrus of control, DFS and HU rats. DGg: the granular layer of the dentate gyrus; h: hilus. Figure S2: Qrt-PCR analysis demonstrated the expression of Sox2 (a) and Cyclin D1(b) mRNA was the same in all groups. n = 4 for each group. Data are shown as median with interquartile range. C: control, DFS: the rats with dynamic foot stimulation during 3-day hindlimb unloading, HU: 3-day hindlimb unloading. Figure S3: Original Western Blots figures.
