# Supplementary material for: Plantar Stimulations during 3-Day Hindlimb Unloading Prevent Loss of Neural Progenitors and Maintain ERK1/2 Activity in the Rat Hippocampus
Source: Life (Basel). 2021 May 17;11(5):449. doi: 10.3390/life11050449 (PMC8157184; doi:10.3390/life11050449)
Supplement: Supplementary file 1 [file life-11-00449-s001.zip › life-1219314-supplementary.pdf]

life

MDPI

Article

Plantar Stimulations during 3-Day Hindlimb Unloading Prevent Loss of Neural Progenitors and Maintain ERK1/2 Activity in the Rat Hippocampus

Anna S. Berezovskaya [1], Sergey A. Tyganov [2], Svetlana D. Nikolaeva [1], Alexandra A. Naumova [1], Boris S. Shenkman [2], and Margarita V. Glazova [1,*]

[1] Sechenov Institute of Evolutionary Physiology and Biochemistry Russian Academy of Sciences, 194223 St. Petersburg, Russia; berezovskaia.annaS@gmail.com (A.S.B.); sveta.nikolaeva@gmail.com (S.D.N.); anaumova07@gmail.com (A.A.N.)

[2] Institute of Biomedical Problems Russian Academy of Sciences, 123007 Moscow, Russia; sentackle@yandex.ru (S.A.T.); bshenkman@mail.ru (B.S.S.)

* Correspondence: mglazova@iephb.ru

Abstract: Adult neurogenesis is a flexible process that depends on the environment and correlates with cognitive functions. Cognitive functions are impaired by various factors including space flight conditions and reduced physical activity. Physically active life significantly improves both cognition and the hippocampal neurogenesis. Here we analyzed how 3-day simulated microgravity caused by hindlimb unloading (HU) or dynamic foot stimulation (DFS) during HU can affect the hippocampal neurogenesis. Adult Wistar rats were recruited in the experiments. The results demonstrated a decrease in the number of doublecortine (DCX) positive neural progenitors, but proliferation in the subgranular zone of the dentate gyrus was not changed after 3-day HU. Analysis of the effects of DFS showed restoration of neural progenitor population in the subgranular zone of the dentate gyrus. Additionally, we analyzed activity of cRaf/ERK1/2 pathway, which is one of the major players in the regulation of neuronal differentiation. The results demonstrated inhibition of cRaf/ERK1/2 signaling in the hippocampus of HU rats. In DFS rats no changes in the activity of cRaf/ERK1/2 were observed. Thus, we demonstrated that the process of neurogenesis fading during HU begins with inhibition of the formation of immature neurons and associated ERK1/2 signaling activity, while DFS prevents the development of mentioned alterations.

Keywords: simulated microgravity; hippocampus; neurogenesis; doublecortin; Ki67; ERK1/2; NR2B

Citation: Berezovskaya, A.S.; Tyganov, S.A.; Nikolaeva, S.D.; Naumova, A.A.; Shenkman, B.S.; Glazova, M.V. Plantar Stimulations during 3-Day Hindlimb Unloading Prevent Loss of Neural Progenitors and Maintain ERK1/2 Activity in the Rat Hippocampus. **2021**, *11*, 449. https://doi.org/10.3390/life11050449

Academic Editor: Fathi Karouia

Received: 27 April 2021
Accepted: 14 May 2021
Published: 17 May 2021

Publisher's Note: MDPI stays neutral with regard to jurisdictional claims in published maps and institutional affiliations.

Copyright: © 2021 by the authors. Licensee MDPI, Basel, Switzerland. This article is an open access article distributed under the terms and conditions of the Creative Commons Attribution (CC BY) license (http://creativecommons.org/licenses/by/4.0/).

Supplementary Materials:

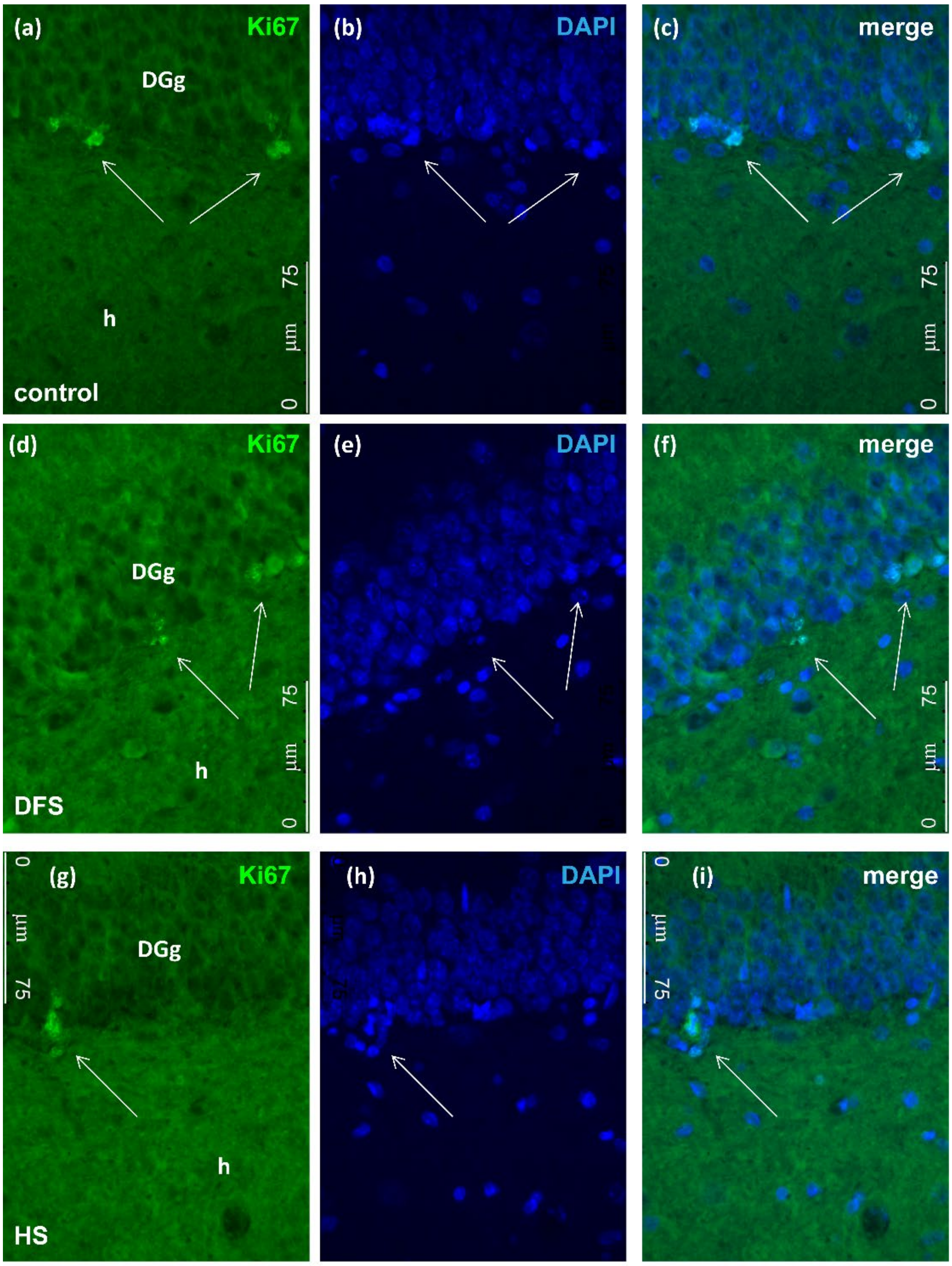

Figure S1. Representative pictures of Ki67 immunostaining (green) in the subgranular zone of the dentate gyrus of control, DFS and HU rats. DGg: the granular layer of the dentate gyrus; h: hilus.

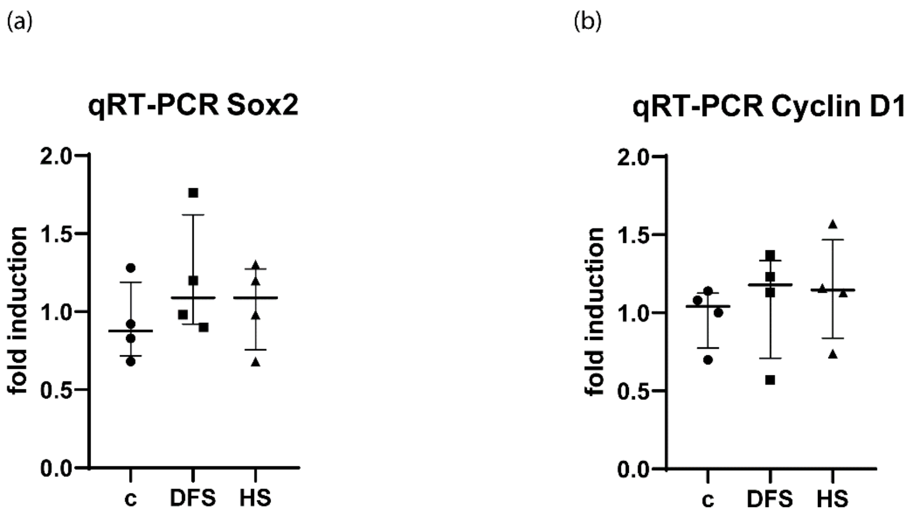

Figure S2. Qrt-PCR analysis demonstrated the expression of Sox2 (a) and Cyclin D1(b) mRNA was the same in all groups. n = 4 for each group. Data are shown as median with interquartile range. C: control, DFS: the rats with dynamic foot stimulation during 3-day hindlimb unloading, HU: 3-day hindlimb unloading.
